# Effect of cavity preparation design and ceramic type on the stress distribution, strain and fracture resistance of CAD/CAM onlays in molars

**DOI:** 10.1590/1678-7757-2018-0004

**Published:** 2018-08-20

**Authors:** Ana Luíza Serralha de Velloso VIANNA, Célio Jesus do PRADO, Aline Aredes BICALHO, Renata Afonso da Silva PEREIRA, Flávio Domingues das NEVES, Carlos José SOARES

**Affiliations:** 1 Universidade Federal de Uberlândia, Faculdade de Odontologia, Departamento de Prótese e Materiais Odontológicos, Uberlândia, Minas Gerais, Brasil.; 2 Universidade Federal de Uberlândia, Escola Técnica de Saúde, Uberlândia, Minas Gerais, Brasil.; 3 Universidade Federal de Uberlândia, Faculdade de Odontologia, Departamento de Dentística e Materiais Odontológicos, Uberlândia, Minas Gerais, Brasil.; 4 Universidade Federal de Uberlândia, Faculdade de Odontologia, Departamento de Oclusão, Prótese Fixa e Materiais Odontológicos, Uberlândia, Minas Gerais, Brasil.

**Keywords:** Ceramics, Computer-aided design, Finite element analysis

## Abstract

**Objective:**

This study aimed to evaluate the effect of the cavity preparation and ceramic type on the stress distribution, tooth strain, fracture resistance and fracture mode of human molar teeth restored with onlays.

**Material and Methods:**

Forty-eight molars were divided into four groups (n=12) with assorted combinations of two study factors: BL- conventional onlay preparation with boxes made from leucite ceramic (IPS-Empress CAD, Ivoclar Vivadent); NBL- conservative onlay preparation without boxes made from leucite ceramic; BD- conventional onlay preparation with boxes made from lithium disilicate glass ceramic (IPS e.max CAD, Ivoclar Vivadent); NBL- conservative onlay preparation with boxes made from lithium disilicate glass ceramic cuspal deformation (µS) was measured at 100 N and at maximum fracture load using strain gauge. Fracture resistance (N) was measured using a compression test, and the fracture mode was recorded. Finite element analysis was used to evaluate the stress distribution by modified von Mises stress criteria. The tooth strain and fracture resistance data were analyzed using the Tukey test and two-way ANOVA, and the fracture mode was analyzed by the chi-square test (α=0.05).

**Results:**

The leucite ceramic resulted in higher tooth deformation at 100 N and lower tooth deformation at the maximum fracture load than the lithium disilicate ceramic (P<0.001). The lithium disilicate ceramic exhibited higher fracture resistance than the leucite ceramic (P<0.001). The conservative onlay resulted in higher fracture strength for lithium disilicate ceramic. Finite element analysis results showed the conventional cavity preparation resulted in higher stress concentration in the ceramic restoration and remaining tooth than the conservative onlay preparation. The conservative onlays exhibited increased fracture resistance, reduced stress concentration and more favorable fracture modes.

**Conclusion:**

Molars restored with lithium disilicate CAD-CAM ceramic onlays exhibited higher fracture resistance than molars restored with leucite CAD-CAM ceramic onlays.

## Introduction

Ceramic restorations are improved because of their increased translucency and light transmission.^1^ Another advantage includes minimal tooth reduction compared with metal ceramics; minimal thermal conductivity; mimic natural dentition because they have desirable properties, including their physical and mechanical properties; excellent biocompatibility to periodontal tissues; reduced plaque accumulation compared with composite resin; and less susceptibility to metal allergies.^2^ ^,^ ^3^ When an indirect restoration is selected as the treatment option for posterior teeth, the clinician must determine the configuration of the cavity preparation.^4^ ^,^ ^5^ Several designs have been proposed for preparing all-ceramic resin-bonded posterior restorations,^6^ ^,^ ^7^ as guided by the particular mechanical and structural characteristics of ceramic restorative materials.^8^

The primary causes of failure of ceramic inlay or onlay restorations are cohesive bulk fractures and marginal deficiencies,^9^ which manifest clinically as marginal discoloration and secondary caries.^10^ Tooth preparation designs for posterior ceramic restorations have been based on traditional cast metal restoration designs, but with more occlusal tooth reduction and with a slightly increased taper.^4^ These preparations may involve the removal of considerable tooth structure.^11^ As more structure is removed, higher tooth strain and lower fracture resistance may occur.^5^ The increased tooth structure loss may increase cuspal flexure, thereby reducing the tooth fracture resistance, or open the restoration-tooth interface.^12^ However, it has been demonstrated that cusp recovery results in fewer failures, likely increasing the longevity of posterior ceramic restorations.^6^ Recently, minimally invasive cavity preparations for posterior indirect restorations were demonstrated to present the benefit of conservation of tooth structure, as well as improved stress distribution.^13^ However, the performance of posterior restoration is also material dependent.^14^ ^,^ ^15^ Due to the continuous advancements in dental ceramics and innovative manufacturing techniques, the following question arises: could traditional preparation guidelines for ceramic onlays be modified in terms of minimally invasive therapy? Several all-ceramic systems, such as leucite and lithium disilicate CAD-CAM systems, have two major recent developments: dentine bonding and stronger all-ceramic crown systems.^16^ Ceramic inlays and onlays can be manufactured in a laboratory or milled chairside from ceramic blocks using CAD/CAM technology.^17^ The restorations prepared with indirect technique with CAD/CAM system in case of more extensive loss of dental structure can be preferred because of their better fracture resistance, esthetic looks, implementation in a single visit, and shorter intraoral working time.^18^ This system shows good clinical performance; however, it depends on the material and its indication in fixed prostheses of one or more elements.^15^ However, the use of these materials is extremely technique sensitive. CAD/CAM ceramic materials are manufactured under optimized conditions, which can minimize voids and volume defects.^17^ ^,^ ^19^ ^,^ ^20^ The fracture rate for CAD/CAM posterior ceramic restorations is suggested to be related to design aspects of the restoration and to the composition of the ceramic.^15^

Fracture resistance tests have been used to predict the failure of ceramic restorations under influence of the preparation design.^21^ Nondestructive experimental methodologies, such as the strain gauge test,^22^ and finite element analysis^23^ ^,^ ^24^ should be combined with conventional mechanical tests to better explain the failure of the ceramic restorations.^22^ ^,^ ^25^ The stresses generated by bite loading cause structural strain; if such stresses become excessive and exceed the elastic limit, structural failure may result.^26^ To the best of the authors’ knowledge, to date, no study has integrally analyzed the failure of minimal cavity preparations for posterior teeth with different ceramic compositions in comparison with conventional cavity preparation designs. Therefore, this study aimed to analyze the biomechanical performance of onlays made from leucite and lithium disilicate-reinforced ceramics in CAD/CAM restorations associated with both conventional cavity preparations and minimal preparations without occlusal and proximal boxes. The null hypothesis was that the ceramic type and cavity preparation design have no effect on the remaining tooth strain, stress distribution, fracture resistance and fracture mode of molars restored with onlays.

## Material and methods

### Teeth selection and cavity preparation

**Figure 1:**
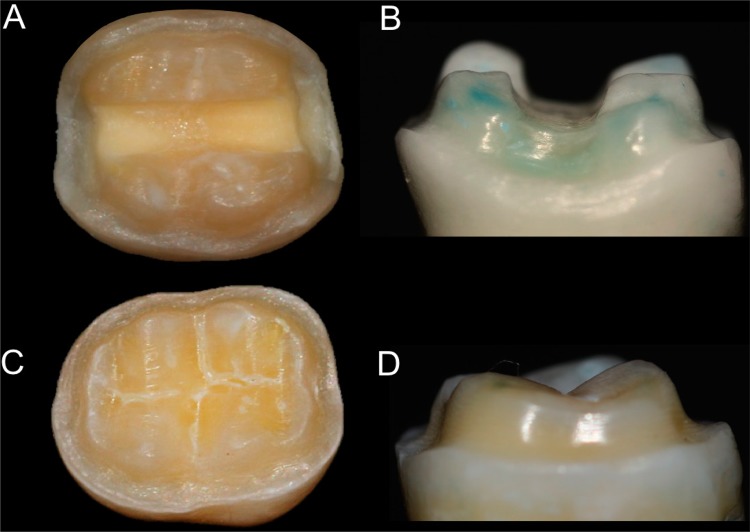
Cavity preparation with occlusal and proximal boxes. (A, B) Cavity with boxes; (C, D) Cavity without boxes

In this *in vitro* study, forty-eight freshly extracted mandibular molars were selected with the approval of the Ethics Committee in Human Research (protocol #307.608). The teeth were selected to have an intercuspal width that fell within a maximum deviation of no more than 10% of the determined mean. The measured intercuspal width varied between 5.2 mm and 6.1 mm. The teeth were embedded in a polystyrene resin (Cristal, Piracicaba, SP, Brazil) up to 2.0 mm below the cervical line to simulate alveolar bone support, and for simulating the periodontal ligament was used a 0.3 mm layer of a polyether impression material (Impregum; 3M ESPE, St Paul, MN, USA ).^27^ The tooth was placed down into a hole in a wooden board, leaving the root in a vertical position perpendicular to the supporting radiographic film, placed over the root and fixed in position with wax. A polystyrene resin was manipulated according to manufacturer and poured into a plastic cylinder. The teeth were removed from the cylinder after resin polymerization, and the wax was removed from the root surface and resin cylinder, simulating the alveolus. The polyether material was placed inside the resin cylinders.^27^ The teeth were cleaned using a rubber cup and fine pumice water slurry and distributed into four groups (n=12) (Figure 1): BL, onlay cavity preparation with proximal and occlusal boxes made from leucite ceramic (IPS Empress CAD, Ivoclar Vivadent AG, Schaan, Liechtenstein); NBL, onlay cavity preparation without proximal and occlusal boxes made from leucite ceramic; BD, onlay cavity preparation with proximal and occlusal boxes made from lithium disilicate glass ceramic (IPS Empress CAD, Ivoclar Vivadent AG, Schaan, Liechtenstein); NBD, onlay cavity preparation without proximal and occlusal boxes made with lithium disilicate glass ceramic.

Before the cavity preparation, x-rays (Timex 70 E, Gnatus, Ribeirão Preto, SP, Brazil) of all the teeth in the buccal and mesial directions were taken. Two different cavity preparation designs with internal rounded angles were defined. For the conventional onlay preparation, the occlusal reduction was 1.5 mm, maintaining the inclination of the cusps using a diamond bur (#2143, KG Sorensen, Barueri, SP, Brazil). The occlusal box was extended by 1.0 mm in depth according to the anatomical characteristics of each tooth, and an overall preparation angle of 6° toward the occlusal aspect was created with a conical flat-end diamond bur (#3131, KG Sorensen, Barueri, SP, Brazil) to produce converging walls to the occlusal. For the preparation of proximal boxes, the same diamond bur was used within 0.5 mm from the gingival margin with an isthmus measuring one third of the buccolingual width. The conservative onlay preparation included only the occlusal reduction, excluding the occlusal and proximal boxes (Figure 1). All the restorations used the minimum thickness of material specified by the manufacturer. All the teeth were prepared using a high-speed handpiece with copious air-water spray, using a cavity preparation machine^28^. This machine consisted of a high-speed handpiece (EXTRA torque 605 C; KaVo do Brasil, Joinville, SC, Brazil) coupled to a mobile base. The mobile base could move vertically and horizontally with three precision micrometric heads (152-389; Mitutoyo Sul Americana Ltda, Suzano, SP, Brazil), attaining a 0.002 mm level of accuracy.

### Ceramic preparation and cementation

An optical impression was made using intraoral digitization (Cerec Blue Cam & MCXL, Dentsply Sirona, Bensheim, Germany) to generate a 3D virtual model. Using CAD, the design of the restoration was created, maintaining the same occlusal anatomy for all the restorations. This image was sent to the CAM, and the biogeneric copy technique was used to obtain the indirect restoration by milling a ceramic block. All CAD/CAM restorations were produced using the CEREC System (CEREC System, Sirona, Germany), and CAD/CAM onlays were fabricated using leucite ceramic (IPS Empress CAD) and lithium disilicate ceramic (IPS E-max CAD). All the restorations were produced and cemented according to the manufacturer’s instructions. The accuracy of each restoration fit was assessed, and adjustments were made when necessary. Leucite-reinforced ceramic onlays were etched with 10% hydrofluoric acid (Condicionador de Porcelanas; Dentsply, São Paulo, SP, Brazil) for 60 s, and lithium disilicate-reinforced ceramic onlays were etched with 10% hydrofluoric acid for 20 s,^29^ followed by silanization using a pre-hydrolyzed silane agent (Angelus, Londrina, PR, Brazil) applied for 60 s and then dried with air spray.^29^ The self-adhesive resin cement (RelyX U200, 3M-Espe, St Paul, MN, USA) was manipulated as recommended by the manufacturer and inserted into the intaglio surface of the ceramic restorations, which were seated in place using digital pressure. Excess luting agent was removed, and after 5 minutes,^30^ ^,^ ^31^ the resin cement was activated using a halogen curing unit (XL 3000; 3M ESPE, St Paul, MN, USA) with 800 mw/cm^2^ checked by using Resin Calibrator (BlueLight, Halifax, NS, Canada), activating in the buccal, lingual, and occlusal directions for 40 s, totaling 120 s for each tooth.

### Strain measurement and fracture resistance tests

Coronal deformation was measured with strain gauges (PA-06-060CC-350L, Excel Sensores, SP, Brazil), which had an internal electrical resistance of 350 X, a gauge factor of 2.1, and a grid size of 21.0 mm^2^. The gauge factor is a proportional constant between electrical resistance variation and strain. The strain gauges were bonded to the lingual surfaces of the ceramic restorations with cyanoacrylate adhesive (Super Bonder; Loctite, São Paulo, SP, Brazil), and the wires were connected to a data acquisition device (ADS0500IP; Lynx Tecnologia Eletrônica, São Paulo, SP, Brazil). The strain gauges were placed in the region in which a finite element model indicated the presence of the highest polymerization stresses. The specimens were subjected to nondestructive axial compressive loading using a metal sphere 6mm in diameter at this orientation and a crosshead speed of 0.5 mm/min^32^ in a mechanical testing machine (DL2000; EMIC) until reaching 100 N, when the first strain value was recorded. Then, the load was applied until failure, and the second strain value was recorded (n=7). The maximum load to cause failure of the sample was recorded (N) for all the samples (n=12). Strain data were transferred to a computer by using specific acquisition signal transformation and data analysis software (AQDADOS 7.02 and AQANALISYS; Lynx, São Paulo, SP, Brazil).

**Figure 2:**
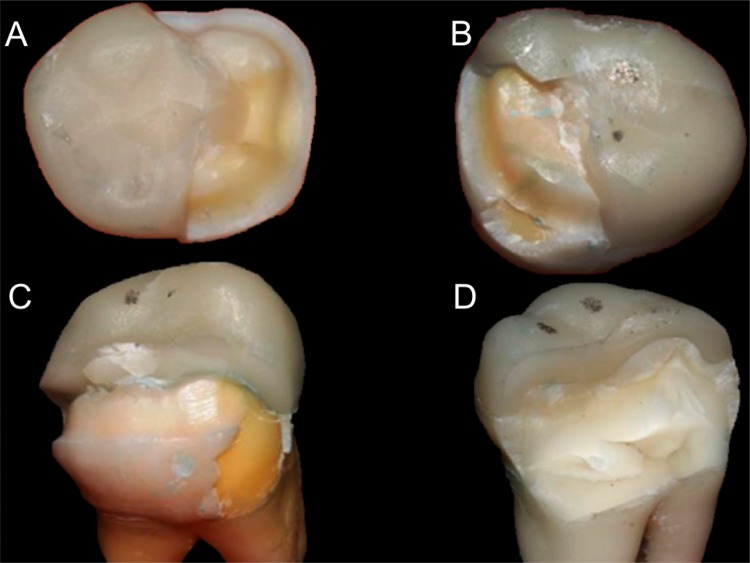
Types of fracture. (A) Type 1, Fractures involving a small portion of the coronal tooth structure; (B) Type 2, fractures involving a small portion of the coronal tooth structure and cohesive failure of the restoration; (C) Type 3, fractures involving the tooth structure, cohesive and/or adhesive failure of the restoration, and root involvement that can be restored in association with periodontal surgery; and (D) Type 4, severe root and crown fracture, necessitating extraction of the tooth

The mode of fracture for each specimen was analyzed under a stereomicroscope (Mitutoyo, Tokyo, Japan) to determine modes of fracture and then assigned to 1 of the 4 categories^33^, as shown in Figure 2.

### Statistical analysis

The data of deformation at 100 N, deformation at maximum fracture load and fracture resistance were tested for normal distribution (Shapiro-Wilk) and the equality of variances (Levene’s test). Two-way analysis of variance (ANOVA) for 2 study factors, ceramic restorations (2 levels: lithium disilicate or leucite ceramic) and cavity preparation (2 levels: conventional or minimally invasive preparations), was performed followed by the Tukey test. The fracture mode data were subjected to the chi-square test. All the tests used a 0.05 level of statistical significance and all statistical analyses were carried out with Sigma Plot version 13.1 (Systat Software Inc., San Jose, CA, USA).

### Finite element analysis – FEA

Buccolingual bidimensional models were created from a longitudinal cut of a sound mandibular molar, simulating the dimensions of each dental structure and of the indirect restoration made by the CAD-CAM system. The stress analysis was performed using MSC.Mentat (preprocessor and postprocessor) and MSC. Marc (solver) software (MSC Software Corporation, Santa Ana, CA, USA). The external outline of the tooth specimen positioned in the polystyrene resin base and simulated periodontal ligament were included in the model. The same experimental conditions used for fracture resistance and strain gauge tests were simulated in FEA. Coordinates were obtained using ImageJ software (public domain, Java-based image processing and analysis software developed at The National Institutes of Health, Bethesda, MD, USA). The mesh was manually created using a four-node isoparametric arbitrary quadrilateral element written for plane strain applications using reduced integration (one integration point - element type 115). The frictional contact was inserted between the metallic sphere and the restored tooth sample. All other interfaces were considered bonded. A dynamic structural analysis was performed. All the materials were assumed to be linear, elastic, isotropic and homogeneous. The mechanical properties were represented by Young’s modulus of elasticity and Poisson’s ratio extracted from the literature (Table 1).^24^ ^,^ ^30^ ^,^ ^31^ ^,^ ^34^ Boundary conditions were defined by the restriction of the movements applied at the external lateral outline and cylindrical specimen support base. Stress distribution analysis was performed by the quantitative association of the main maximum stresses by the modified von Mises criteria.

**Table 1:** Mechanical properties of isotropic structures

Structure	Elastic Modulus (MPa)	Poisson Ratio	References
Enamel	84.100	0.20	37, 40
Dentin	18.600	0.31	37, 40
Pulp	2.0	0.45	37, 38
Periodontal ligament	50.0	0.45	37, 38
Polystyrene resin	13.500	0.31	42
Lithium disilicate ceramic	96.000	0.25	36
Leucite ceramic	65.000	0.23	36
Resin cement	8.600	0.30	36, 40

## Results

### Coronal Deformation (CD)

The tooth deformation values (strain) for the two ceramic restorations and the two cavity preparations at 100 N are shown in Table 2. Two-way ANOVA showed ceramic type factor (P=0.005) had significant effects on tooth deformation; however, the cavity preparation factor (P=0.426) interaction between the two study factors had no significant effect (P=0.258). The Tukey test showed leucite ceramic restorations had a significantly higher deformation than lithium disilicate ceramic restorations, irrespective of the cavity preparation design (P<0.001). Both cavity preparations had similar deformation, irrespective of the type of ceramic restoration (P=0.942).

**Table 2:** Coronal deformation (µS) measured by strain gauges (n=7 teeth)

Ceramic Type	Coronal deformation (µS)			
	100 N		Maximum fracture load	
	Cavity preparation without box	Cavity preparation with box	Cavity preparation without box	Cavity preparation with box
Lithium disilicate ceramic	31.7±5.6^Aa^	34.2±10.8^Aa^	1141.0±155.4^Ba^	1151.9±134.9^Ba^
Leucite ceramic	58.1±17.5^Ba^	48.8±7.9^Ba^	695.4±137.6^Aa^	749.5±68.1^Aa^

Different uppercase letters in columns indicate the ceramic type for each cavity preparation design and load condition; lowercase letters in rows indicate the cavity preparation design for each ceramic and load condition (P<0.05)

The coronal deformation values (strain) for the two ceramic restorations and the two cavity preparations at the maximum fracture load are shown in Table 2. Two-way ANOVA showed the ceramic type (P=0.020) had a significant effect on fracture resistance; however, the cavity preparation (P=0.426) and the interaction between the two study factors had no significant effect (P=0.258). The Tukey test showed the lithium disilicate ceramic restorations exhibited significantly higher deformation than leucite restorations (P=0.029). Both cavity preparations had similar deformation, irrespective of the type of ceramic restoration (P=0.258).

### Fracture resistance and fracture mode

The fracture resistances in N for the two ceramic restorations and the two cavity preparations are shown in Table 3. Two-way ANOVA revealed that the ceramic restoration (P<0.001), the cavity preparation (P<0.001) and the interaction between the two study factors (P=0.018) had significant effects on fracture resistance. The Tukey test showed the presence of a box had no significant effect for leucite ceramic restorations (P=0.375); however, the presence of a box in lithium disilicate ceramic restorations significantly reduced the fracture resistance (P<0.001). The lithium disilicate ceramic restorations had significantly higher fracture resistance than leucite ceramic restorations (P<0.001), irrespective of the cavity preparation.

**Table 3:** Fracture resistance (N) measured by the axial compression test (n=12 teeth)

Ceramic Type	Fracture Resistance – N	
	Cavity preparation without box	Cavity preparation with box
Lithium disilicate ceramic	3099.1±757.3^Aa^	2108.6±476.9^Ab^
Leucite ceramic	1794.9±516.3^Ba^	1591.3±414.6^Ba^

Different uppercase letters in columns indicate the ceramic type for each cavity preparation design; lowercase letters in rows indicate the cavity preparation design for each ceramic (p<0.05)

**Figure 3:**
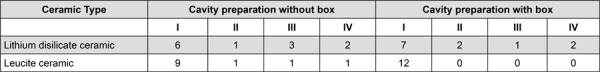
Fracture mode distribution (n=12 teeth)

The fracture mode distributions are shown in Figure 3. The chi-square test showed the lithium disilicate ceramic resulted in a more severe fracture mode, irrespective of the type of cavity preparation.

Fracture modes: I, fractures involving a small portion of the coronal tooth structure; II, fractures involving a small portion of the coronal tooth structure and cohesive failure of the restoration; III, fractures involving the tooth structure, cohesive and/or adhesive failure of the restoration, and root involvement that can be restored in association with periodontal surgery; and IV, severe root and crown fracture, necessitating extraction of the tooth

### Finite element analysis

**Figure 4:**
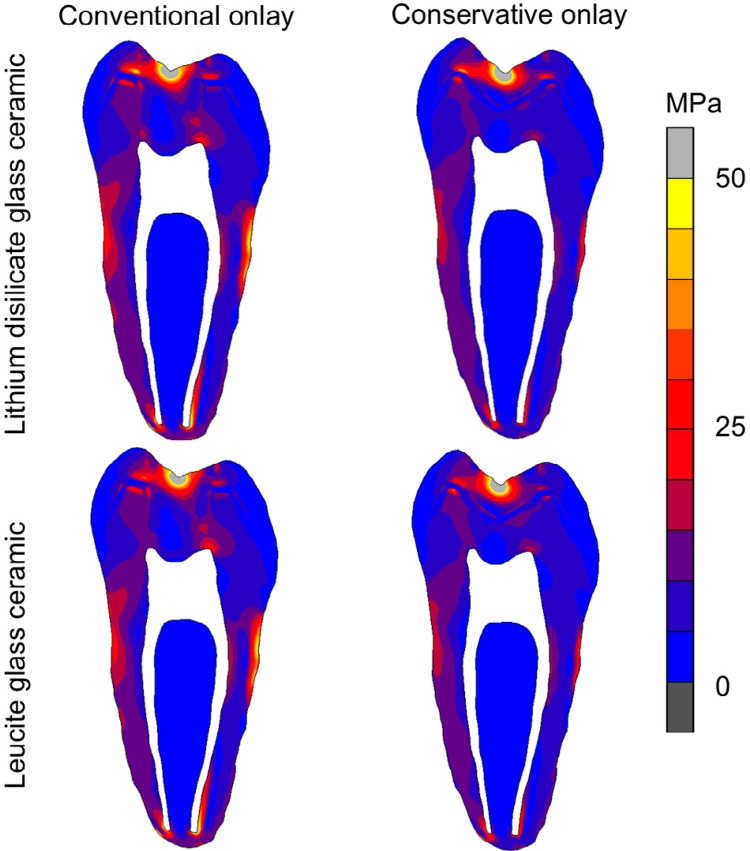
Modified von Mises stress distributions for all groups at 100 N. (A) Conventional onlay/ lithium disilicate glass ceramic; (B) Conventional onlay/leucite glass ceramic; (C) Conservative onlay/lithium disilicate glass ceramic; (D) Conservative onlay/leucite glass ceramic

Modified von Mises stress distributions for all the groups at 100 N are shown in Figure 4. The type of cavity preparation influenced the stress distribution and intensity more than the type of ceramic. The cavity preparation with an occlusal box resulted in higher stresses at the ceramic restoration and higher remaining tooth structure than cavity preparations without occlusal box. The lithium disilicate ceramic restorations resulted in a slightly higher stress concentration in the ceramic than leucite ceramic restorations.

## Discussion

This study investigated the influence of ceramic type and cavity preparation design on the tooth remaining deformation, stress distribution, fracture resistance and fracture mode of molar restored with ceramic onlays. The results showed that the lithium disilicate ceramic had better performance than leucite ceramic onlay and that conservative cavity preparation without occlusal and proximal boxes is the best choice for improving biomechanical performance of posterior ceramic onlays. Therefore, the null hypothesis was rejected.

To minimize the discrepancy between experimental assessments and clinical failures, different methods have been used, such as the association of tooth remaining deformation, fracture resistance tests, fracture mode analysis and finite element analysis.^22^ ^,^ ^25^ ^,^ ^35^ ^,^ ^36^ *In vitro* tests are the primary methods used to investigate the fracture strength of restorations; however, the different methodologies used in different studies, such as the mode and direction of load application, crosshead speed, fracture mode, and root embedding, may result in different outcomes, making any comparison difficult.^21^ Tooth fracture is defined by the moment when stress intensity exceeds a critical value prompting rupture.^32^ The periodontal ligament plays an important role in this failure process, because it can deform and accommodate the tooth in the alveolus, which alleviates stress in the cervical region of the tooth. In this experiment, a polyether impression material was used with polystyrene resin to simulate more realistic fractures observed clinically.^27^ Other important aspect is the speed employed on the fracture resistance test, structures with ductile characteristics tend to be brittle when submitted to higher crosshead speed load applications.^32^ To simulate the tooth fracture with compressive loading, crosshead speeds of 0.5 were used in this study, which allows a better stress distribution inside the restored tooth.^32^ In the biomechanical analysis of tooth structures and restorative materials, destructive mechanical tests used to determine fracture resistance are important means of analyzing tooth behavior in situations of high intensity load application. However, these tests do show limitations with regard to obtaining information about the internal behavior of the tooth-restoration complex. For a more precise response, the combination of experimental nondestructive methodologies, such as strain-gauge test,^22^ ^,^ ^25^ and finite element analysis, with conventional mechanical tests, seems appropriate.^1^ ^,^ ^22^ ^,^ ^25^ ^,^ ^36^ ^-^ ^38^ The association of experimental tests and computational analysis, characterizing the fracture of a restorative material or tooth structure and the strain/stress behavior, provides important data to facilitate the improvement of restorative procedures.^39^ In this study, a non-linear finite element analysis using modified von Mises stress was performed for comparing different models. Modified von Mises equivalent stress expressed the stress conditions, using compressive-tensile strength. Stresses in three dimensions were integrated into one scalar value by using a modified von Mises criterion to represent the overall stress condition that could be used to show areas with most critical stress concentrations.^25^

Many variables can affect the fatigue and fracture behavior of all-ceramic restorations, including the dimensions of core and veneer materials, inherent or processing flaws within the materials, and preparation design.^11^ ^,^ ^21^ In this study, in order to control the thickness of the ceramic restorations, the CAD/CAM method was used. Ceramic thickness and geometry of cavity preparations are key factors that influence the clinical longevity of all-ceramic restorations.^4^ Enamel is a natural brittle structure that covers the crown of the tooth, it is under layered by dentin, which has ductile behavior. The enamel and dentin are integrated by uniform transition without sharped angle. The conservative cavity preparation confirmed that the uniform thickness of the brittle material is important for stress/strain transferring between structures with different elastic moduli.^37^ ^,^ ^38^ The finite element analysis showed the cavity preparation influenced the stress concentration more than the type of ceramic. Conventional onlay models, with occlusal and proximal boxes, showed higher concentrations of stress in the ceramic restoration and in the remaining tooth structure than the conservative onlay. This may be explained by the larger amount of tooth structure removed from the occlusal surface, which creates more sharp angles that may concentrate stress. Additionally, the ceramic thickness increasing impacted on the stress concentration level inside of the material.

The leucite ceramic resulted in greater tooth deformation than that of lithium disilicate ceramic at 100 N, irrespective of the type of cavity preparation. A load applied to an object causes the stress concentration and structural strain. The direct and linear relationship between stress and strain is primarily promoted by the elastic modulus, an important mechanical property that is fundamental to understand the biomechanical behavior of materials and their relationships.^40^ The elastic modulus of the restorative lithium disilicate is greater than that of the leucite ceramic. The stiff material tends to concentrate stress inside the material, reducing the stress transfer to the remaining tooth structure. A recent study accessed the database of an industry-scale machining center in Germany and obtained information on 34,911 CAD/CAM all-ceramic posterior restorations, showed the fractures rates over a period of 3.5 y, reported that the lithium disilicate showed significantly better performance than the leucite-based Empress CAD for onlays and inlays, highlighting the role of the microstructure in the fracture process.^15^

When the load is within the elastic limit of the restored tooth, the structural integrity is not affected. When the tooth structure is removed, more cusp strain is observed, requesting more of the interfaces, and then may reduce the fracture resistance.^12^ In the presence of the higher levels of the stress concentration factors and high load applied on the occlusal surface, the concentrated stress may result in crack formation and propagation, causing fracture and structural failure. Although the IPS e-max CAD has greater elastic modulus and stiffness than IPS empress CAD,^17^ at a maximum fracture load, the samples restored with lithium disilicate exhibited greater tooth structure deformation than the samples restored with leucite. This may be because the load exceeded the elastic limit of the resistance of the remaining tooth structure. The maximum load, which can reach values higher than 4500 N, exceeds the upper limits of a normal occlusion. Additionally, the stress generated was most concentrated within the ceramic material and could initiate crack formation and propagation, resulting in the cohesive fracture of the ceramic material. This may explain why most of the samples exhibited fractures of ceramic restorations before failure of the remaining tooth structure.

In this study, the lithium disilicate ceramic groups had significantly higher fracture resistance than the leucite ceramic restoration groups, irrespective of the type of cavity preparation. This may be due to the higher elastic modulus and fracture strength.^17^ The disilicate ceramic can support higher load, absorbing greater amounts of energy inside the ceramic material before fracture. This aspect is very important in the new paradigm that determines conservative occlusal reduction. Therefore, for occlusal reconstruction in patients with bruxism, lithium disilicate may be preferable. Analyzing the fracture modes in addition to fracture resistance is important. The findings of this study may be explained by the higher stiffness of lithium disilicate, which reaches the yield strength and leads to the fracture of the remaining tooth structure. The lesser deformation of lithium disilicate is caused by the higher elastic modulus and, therefore, leads to support greater deformation of the remaining tooth structure, resulting in a higher percentage of complex fracture. The maximum preservation of healthy tooth structure and the use of restorative materials with mechanical properties similar to dental structure may promote a greater longevity of the tooth-restoration complex.

## Conclusions

Within the limitations of this *in vitro* study, the following conclusions can be drawn:

1. Ceramic onlays with conservative preparation without occlusal and proximal boxes demonstrated better biomechanical performance than conventionally prepared ceramic onlay restorations;
2. The thickness of ceramic restorations influenced the stress concentration, in which a more homogeneous thickness promoted a better stress distribution;
3. Conservative preparations resulted in higher fracture resistance in molars restored with lithium disilicate CAD-CAM ceramic onlays;
4. Molars restored with lithium disilicate CAD-CAM ceramic onlays exhibited higher fracture resistance than molars restored with leucite CAD-CAM ceramic onlays;
5. The association of the strain-gauge test with fracture resistance, fracture mode and finite element analysis provides a better explanation of the failure process of posterior ceramic restorations.
